# Understanding the Long-Lasting Effects of Fetal Nutrient Restriction versus Exposure to an Obesogenic Diet on Islet-Cell Mass and Function

**DOI:** 10.3390/metabo11080514

**Published:** 2021-08-04

**Authors:** Stephanie E. O’Hara, Kelly M. Gembus, Lisa M. Nicholas

**Affiliations:** The Robinson Research Institute, Adelaide Medical School, University of Adelaide, Adelaide, SA 5005, Australia; stephanie.ohara@adelaide.edu.au (S.E.O.); kelly.gembus@adelaide.edu.au (K.M.G.)

**Keywords:** pancreatic beta-cells, intrauterine growth restriction, maternal obesity, developmental programming

## Abstract

Early life represents a window of phenotypic plasticity. Thus, exposure of the developing fetus to a compromised nutritional environment can have long term consequences for their health. Indeed, undernutrition or maternal intake of an obesogenic diet during pregnancy leads to a heightened risk of type 2 diabetes (T2D) and obesity in her offspring in adult life. Given that abnormalities in beta-cell function are crucial in delineating the risk of T2D, studies have investigated the impact of these exposures on islet morphology and beta-cell function in the offspring in a bid to understand why they are more at risk of T2D. Interestingly, despite the contrasting maternal metabolic phenotype and, therefore, intrauterine environment associated with undernutrition versus high-fat feeding, there are a number of similarities in the genes/biological pathways that are disrupted in offspring islets leading to changes in function. Looking to the future, it will be important to define the exact mechanisms involved in mediating changes in the gene expression landscape in islet cells to determine whether the road to T2D development is the same or different in those exposed to different ends of the nutritional spectrum.

## 1. Introduction

Before the ‘developmental origins of adult health and disease’ paradigm emerged, it was widely accepted that type 2 diabetes (T2D) was wholly genetically determined [1]. However, in 1991, upon linking birth weight records to glucose tolerance in men, a relationship was observed between lower weight at birth and a higher risk of T2D in adult life; men with the lowest weight at birth were six times more likely to develop T2D when compared to the heaviest [2]. This association has since been replicated in a number of epidemiological studies [3], is present in both men and women [4] and persists even after adjusting for ethnicity, childhood socioeconomic status and adult lifestyle factors [5].

In 1992, the ‘thrifty phenotype’ hypothesis was put forward by Hales and Barker to explain the association between poor fetal growth and increased T2D risk in adult life. This hypothesis posits that when faced with poor nutrition, the fetus adapts to their environment by selectively protecting brain growth at the detriment of other tissues and organs. This includes impaired development of the endocrine pancreas, leading to abnormal pancreatic beta-cell mass/function and reduced capacity for insulin secretion, which persists into adult life [1]. Indeed, offspring who were exposed to periods of famine whilst in utero are at a higher risk of T2D in adult life (reviewed in [6]).

In 1998, Hattersley and Tooke put forth the ‘fetal insulin’ hypothesis as an alternative explanation for the association between low birth weight and risk of T2D. This hypothesis proposes that both phenotypes are the result of a genetic predisposition for reduced insulin secretion and action leading to decreased growth in fetal life and a decreased capacity to secrete and respond to insulin and, therefore, higher T2D risk [7,8]. Support for the ‘fetal insulin’ hypothesis emerged from genome-wide association studies showing that a number of T2D risk loci are associated with lower birthweight. Furthermore, the strongest associations are at loci that primarily affect beta-cell function (reviewed in [8]). However, it has since been argued that genetic susceptibility does not solely account for the relationship between low birth weight and T2D; in a study of monozygotic twins discordant for T2D, the diabetic twin had a lower birth weight compared to their genetically identical non-diabetic co-twin [9].

Over the last two decades, the increasing number of studies reporting incidence of high birth weight (especially given the higher prevalence of maternal obesity/gestational diabetes in more contemporary cohorts) have provided additional clarity regarding the relationship between low versus birth weight and T2D risk; systematic review and meta-analysis have shown that babies born with either low (<2.5 kg) or high (>4.5 kg) birth weight are most at risk for T2D in later life [3,10]. This is particularly worrying given that in many populations, nearly half of women of child-bearing age are either overweight or obese [11]; not only are these women twice more likely to have a baby with high birth weight [12], adult children of overweight and obese mothers also have a 1.4- and 3.5-fold higher incidence of T2D, respectively, compared to children of normal weight mothers [13].

Similar to fetal undernutrition, it remains unresolved whether genetic predisposition [14,15] or the intrauterine/early life environment [13] have a larger part to play in driving the relationship between maternal obesity and offspring T2D risk. However, given that defective pancreatic beta-cells account for most (if not all) forms of diabetes, a large number of studies have been undertaken to elucidate the impact of intrauterine growth restriction (IUGR) as well as sub-optimal maternal nutrition (in the form of undernutrition or exposure to foods that are high in fat and sugar with low nutritional value) on islet morphology and beta-cell function in the offspring.

In this review, we consolidate the evidence across various small and large animal models of restricted fetal growth and maternal caloric excess and discuss both the differences and similarities in islet-specific phenotypes that arise in offspring exposed to these contrasting maternal environments. We also evaluate the proposed mechanisms driving beta-cell dysfunction to determine whether these early life insults result in specific vulnerabilities within beta-cells, leading to an increased risk of T2D. Finally, we share some ideas that we believe are important areas of future research in the field.

## 2. An Overview of Animal Models of Restricted Fetal Growth and Exposure to Maternal Caloric Excess

Animal models have been indispensable for understanding the potential causative mechanisms underlying the relationship between a compromised early life environment and future risk of T2D. The use of genetically identical rodent strains has enabled researchers to dissect the relative contributions of specific components of the maternal (and hence intrauterine) milieu on offspring phenotypes. Furthermore, given that it is not possible to obtain longitudinal samples from human offspring and that the nutritional history of human islet donors are unknown, mechanistic understanding of altered islet function and mass in affected offspring have solely relied on animal models.

### 2.1. Animal Models of Restricted Fetal Growth

Inadequate nutrition during fetal development, usually the result of maternal undernutrition and placental insufficiency, leads to IUGR and low birth weight. Given that amino acid supply is disrupted in growth restricted babies [16,17] and that amino acids are critically important for insulin production, Hales and Barker postulated that defects in the development of fetal pancreatic beta-cells are due to maternal amino acid deficiency [1]. Thus, a number of studies have investigated the impact of IUGR on offspring islets by feeding rodents an isocaloric diet with approximately 60% less protein content compared to controls [18,19,20,21,22,23,24,25,26], which leads to ~10% reduction in offspring birth weight followed by a period of catch-up growth [21,27,28,29]. This has been a core model used in the field. It should be noted, however, that not all models of maternal protein restriction result in an IUGR phenotype in the offspring [20] for reasons that remain unknown. Another well-characterized rodent model of IUGR that has been used to study its effect on islet development is uterine artery ligation. This model induces uteroplacental insufficiency, therefore, limiting the supply of nutrients and oxygen to the fetus leading to a 10–20% decrease in body weight [30,31,32,33,34,35,36,37].

A similar effect has also been achieved in a sheep model of IUGR by surgically removing most of the sites of placentation prior to pregnancy [38,39]. In contrast, a much more severe effect on offspring birth weight (40% reduction) is observed when pregnant sheep are exposed to elevated ambient temperatures in mid to late gestation [40,41,42,43,44]. Despite differences in the method used to induce IUGR and the timing and duration of insult, impaired glucose tolerance and T2D is observed in the offspring across most models, which is in line with findings in human cohorts.

### 2.2. Animal Models of Maternal Caloric Excess

Given the rising rate of overweight and obesity amongst women of child-bearing age, a number of animal models have been developed involving excess maternal caloric intake during pregnancy and lactation. Japanese Macaques fed a high-fat diet has been used as a model that most closely mimics maternal obesity in humans [45,46,47,48] given that dams typically give birth to a single offspring at a time and macaque colonies are genetically heterogeneous. Islets from the Japanese Macaque are also similar to humans in terms of structure, insulin secretion and the expression of endocrine hormones and key transcription factors [49]. Another large animal that has been used to model maternal obesity is sheep. In addition to having only one to three offspring per pregnancy, islet development in sheep is also similar to humans. However, rather than feeding ewes a diet high in fat, obesity is achieved using the same diet as controls but ewes are fed at 150% of their energy requirements [50,51].

Most rodent models of maternal caloric excess involve consumption of a diet that is either high in fat or high in both fat and sugar (with the latter being more reflective of an obesogenic Western diet in humans). Fat content varies across studies ranging from 25 to 50% kcal and is mainly composed of saturated fats. In general, these models result in a maternal phenotype of obesity and impaired glucose tolerance during pregnancy in studies where mice are fed the obesogenic diet for 6–10 weeks before mating (reviewed in [52]). To elucidate the specific maternal characteristics associated with an obese pregnancy in mediating altered islet phenotype in the offspring, one study used the agouti viable yellow (Avy) mouse to model maternal obesity without impaired glucose tolerance [53]. The agouti signaling molecule antagonizes satiety signaling. Thus, Avy/a mice develop obesity when provided ad libitum access to standard chow due to hyperphagia. In contrast, mice carrying a silent allele are lean and metabolically healthy [54,55,56].

In contrast to animal models of restricted fetal growth where metabolic dysfunction is observed in the offspring across most models, the differences in diet composition as well as the duration and timing of maternal over-feeding has led to metabolic phenotypes that are not always entirely consistent between studies (reviewed in [52]). Despite this, it is clear from the numerous studies published across a number of different species that exposure to an obesogenic intrauterine environment predominantly results in metabolic dysfunction including obesity and impaired glucose tolerance in the offspring in adult life (reviewed in [57,58]).

## 3. The Impact of a Compromised Early Life Environment on Beta-Cell Morphology and Function

### 3.1. The Impact of Restricted Nutrition versus Caloric Excess on Islet Morphology

**Figure 1:**
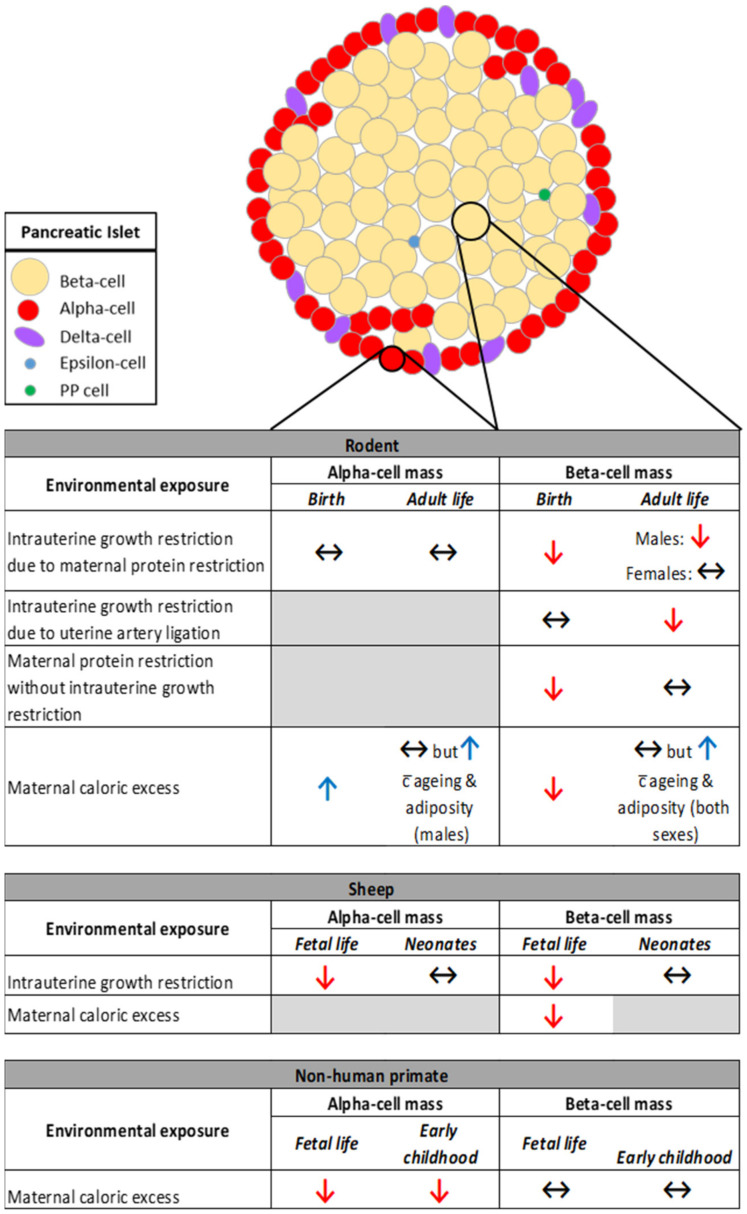
The impact of exposure to restricted nutrition versus caloric excess in rodent, sheep and non-human primate on offspring alpha- and beta-cell mass. *Red arrow: Decreased; Blue arrow: Increased; Black arrow: No change; Gray shading indicates data is not available*.

Compromised nutrition during development, irrespective of whether in the form of maternal nutrient restriction (low-protein diet) or exposure to an obesogenic environment, results in reduced beta-cell mass at birth in rodents [18,59,60] (Figure 1). This is despite a divergent birth weight phenotype between the models; a maternal low-protein diet results in reduced birth weight whereas exposure to maternal obesity has no impact on offspring birth weight [59]. In the case of offspring exposed to a maternal low-protein diet, diminished beta-cell mass is due to reduced proliferation and increased apoptosis of beta-cells in fetal life associated with reduced levels of growth factors [60,61,62,63]. Whether the same occurs in offspring exposed to maternal obesity has not been investigated.

In adult life, beta-cell mass remains reduced in the offspring of protein-restricted dams [18,63]. Importantly, the relationship between lower birth weight (<3 kg versus >3 kg) and reduced beta-cell area in adult life is also observed in humans [64]. In the case of maternal obesity, offspring beta-cells have the ability, to a certain extent, to compensate for changes in body weight and adiposity; beta-cell mass is unchanged in lean, metabolically healthy male and female adult offspring of obese dams [65] but increases with increasing age and adiposity in both sexes [59,66,67] (Figure 1).

In contrast to a maternal low-protein diet, IUGR in rodents induced by uterine artery ligation (which leads to an acute decrease in fetal nutrients and oxygen near term) does not impact on offspring beta-cell mass at birth despite a ~15% reduction in birth weight [68]. There is, however, an eventual decline in beta-cell mass from seven weeks of age into adult life that occurs before the onset of hyperglycemia and is not associated with increased beta-cell apoptosis [68]. This suggests that irrespective of whether fetal exposure to restricted nutrition is chronic or acute, beta-cells are particularly sensitive to IUGR and, importantly, these changes are permanent; there is no recovery of beta-cell mass in offspring despite the strong replicative potential of newborn beta-cells and the normalization of body weight [18,63]. Findings from Theys and colleagues suggest that perhaps male offspring are particularly susceptible; beta-cell mass was unaffected in adult female offspring exposed to maternal protein restriction [21]. Interestingly, when maternal protein restriction is not accompanied by IUGR, whilst the fraction of beta-cells is still reduced in the newborn this is recovered by the time both male and female offspring reach adulthood [20]. Taken together, these findings highlight the complexities involved in understanding how compromised nutrient availability during discrete windows of development can lead to longer term changes in beta-cell mass.

Similar to what is observed in rodents, beta-cell mass is reduced in fetal sheep in late (90% of) gestation in response to both IUGR [44] and maternal obesity [51] with the former due to decreased proliferation and the latter increased apoptosis. In contrast to rodents, however, beta-cell mass is normalized in growth restricted lambs by two weeks of age [69] (Figure 1) suggesting that sheep beta-cells have increased plasticity despite nutrient restriction in utero. While the longer term impact of exposure to maternal obesity on beta-cell mass in sheep offspring has not been investigated, acute insulin response to glucose is decreased in these offspring during a glucose tolerance test in adult life [70].

In a non-human primate model, beta-cell mass is comparable between offspring exposed to either a maternal Western-style or control diet during pregnancy and lactation. This was observed in both fetal life [46] and early childhood (three years of age) [45] (Figure 1). We speculate that one of the reasons beta-cell mass is not impacted in these young offspring is that maternal metabolic dysfunction in response to an obesogenic diet is less pronounced in Japanese Macaques compared to rodents. This is the case even for those with a higher sensitivity to weight gain and insulin resistance in response to a high-fat diet [71].

In contrast to beta-cell mass, alpha-cell mass is unchanged in growth-restricted rodent offspring in late gestation [63] and at birth [72]. There is, however, a decrease in alpha-cell mass one week after birth but this is normalized by two weeks of age and maintained into adult life [72] (Figure 1). Alpha-cell mass is differentially impacted by IUGR in sheep offspring; it is reduced in late gestation due to decreased pancreas weight (the percentage of glucagon-positive area is not different between groups) [44]. Like beta-cell mass, alpha-cell mass is normalized in growth restricted lambs by two weeks of age given that pancreas weight is no longer different between the groups [69] (Figure 1). A recent study in humans also found no association between birthweight and alpha-cell mass in adult life [64].

Maternal high-fat feeding throughout pregnancy leads to increased alpha-cell volume in one-day-old rodent offspring [73]. This increase is not maintained in lean, metabolically healthy adult offspring of obese dams [65] but similar to beta-cells, alpha-cell mass is increased in response to increasing adiposity and age in male [59,74] but not female [67] offspring (Figure 1). Alpha-cell mass in sheep offspring exposed to maternal obesity has not been investigated. The alpha-cell phenotype observed in rodent models of maternal obesity/high-fat feeding are in contrast to findings in Japanese Macaques where a maternal Western-style diet leads to decreased alpha-cell mass in fetal life [46] (despite minimal effect on the overall physiological phenotype of the fetus), which still persists at three years of age in both male and female offspring [45] (Figure 1). Whether these changes impact on offspring T2D risk is unclear given that there is no consensus on how alpha-cell mass is impacted in response to T2D in both rodents and humans [75,76,77]. It should also be noted that whether the population of other endocrine-cell types in offspring islets is affected by exposure to either restricted nutrition or caloric excess has not been investigated.

### 3.2. The Impact of Restricted Nutrition versus Caloric Excess on Beta-Cell Function

Insulin producing beta-cells play a central role in the pathogenesis of T2D; the inability of beta-cells to adapt and compensate for increased insulin demand, for example, which develops as a consequence of insulin resistance in ageing or obesity, leads to T2D [78]. In contrast, sustained beta-cell adaptation is capable of preventing T2D, even in the face of severe insulin resistance [79]. In addition to having more beta-cells, plasticity of beta-cell function also contributes to the compensatory increase in insulin output. Moreover, given that impaired beta-cell function is an early feature of T2D pathogenesis [80], it is important to determine whether beta-cell function is impacted in offspring exposed to restricted nutrition versus caloric excess. This will enable us to better understand the etiology of T2D in response to compromised nutrition during development. Unfortunately, in contrast to the number of studies that have determined the impact on beta-cell mass, only a handful of studies have directly investigated beta-cell function in the offspring.

In rodent models, both IUGR (maternal protein restriction, uterine artery ligation) and exposure to maternal diet-induced obesity is associated with impaired glucose-stimulated insulin secretion in islets of adult offspring [60,63,68,81]. This is perhaps not surprising given that in most studies, islet function was assessed in offspring with increased adiposity and impaired glucose tolerance. Furthermore, it may also explain why glucose intolerance is present in offspring exposed to maternal high-fat feeding despite having greater beta-cell mass [82]. Overall, severity of the insulin secretion phenotype varies across models with the most pronounced effect seen in adult offspring that were growth restricted in utero; the insulin secretory response to glucose is virtually absent in these offspring [63,68]. Surprisingly, islets from offspring exposed to maternal protein restriction but who were not growth restricted at birth also displayed impaired glucose-stimulated insulin secretion and reduced islet insulin content [20]. Similar to the observations made in maternal obesity-exposed mice, these offspring have impaired glucose tolerance despite increased beta-cell mass [20]. It should be noted that these studies did not compare effects between male and female offspring.

In Japanese Macaques, although the effects of a maternal high-fat diet on offspring islet morphology are subtle, both first- and second-phase insulin secretion was increased in response to elevated glucose levels when compared to controls. This was found in very young (three years old), metabolically healthy offspring [45], however, it is unknown whether this was present in only one or both sexes.

Only a small number of studies have compared the sex-specific impact of compromised nutrition during development on offspring beta-cell function. These studies found that in response to either IUGR by maternal protein restriction or to maternal diet-induced obesity, islets from female offspring secrete more insulin for a given glucose load compared to male offspring. This occurs in the absence of increased insulin demand i.e., impaired glucose tolerance and increased adiposity in both male and female offspring [21,65,66]. Furthermore, in mice, only adult male offspring exposed to maternal obesity develop T2D (at six months of age) despite increased adiposity in both sexes [83]. Therefore, we speculate that at least in the case of exposure to an obesogenic diet during pregnancy and lactation, beta-cells from female offspring are primed to better cope with increasing insulin demand in adult life. However, there may be a limit to this ability especially in the face of both ageing and obesity [81]. Curiously, when maternal obesity is modelled using Avy mice that are normoglycemic during pregnancy, female offspring are more vulnerable to metabolic disease with age compared to male offspring. Aged females developed glucose intolerance and had reduced glucose-stimulated insulin secretion following nearly nine months of high-fat feeding in postnatal life [53]. This is in contrast to another study, which found that the latent predisposition to metabolic disease in offspring of Avy dams was more prominent in males who developed glucose intolerance and insulin resistance after only three weeks on a high-fat diet [55]. The reason(s) for the contrasting findings between studies is unknown.

Findings are less consistent in relation to the impact of maternal obesity on basal insulin secretion. Furthermore, there is variability in what constitutes ‘basal’ glucose levels with studies using either lower (1, 2, and 2.8 mM glucose) or slightly higher (5.5 mM glucose) concentrations. We have previously shown that islets from metabolically healthy female offspring of obese dams have a slight but significant increase in insulin secretion compared to controls when incubated in 2.8 mM glucose [65]. In studies performed on offspring in which weight gain, increased adiposity or glucose intolerance is observed, whilst one study found that islets from male offspring exposed to maternal obesity secreted almost double the amount of insulin in response to 1 and 5.5 mM glucose [84], others found no impact on basal insulin secretion (in both males and females) [60,63,81]. Meanwhile, Zambrano and colleagues found that insulin secretion in response to 5.5mM glucose was decreased in both male and female offspring [67]. These inconsistencies in study outcomes suggest that basal insulin secretion may be particularly sensitive to even slight differences (which are likely to be present between studies) in either the pre- or postnatal environment or their interaction.

## 4. Similarities and Differences in the Mechanisms Identified in Offspring Exposed to Restricted Nutrition versus Caloric Excess

Thus far, studies have taken a targeted approach to identifying mechanisms that are contributing to compromised islet function and mass in offspring exposed to malnutrition, focusing on genes and pathways with known effects on beta-cell function. Importantly, the impact is observed even in very young offspring and persists as they age suggesting that these mechanisms are programmed by the intrauterine environment and may, therefore, be the central driver of T2D risk in affected offspring.

### 4.1. The impact of Restricted Nutrition versus Caloric Excess on Pdx1 Expression

Pancreatic and duodenal homeobox 1 (Pdx1) is a transcription factor that is critical for the regulation of pancreatic development and beta-cell differentiation. Pdx1 levels are reduced in human islets from T2D donors [85] and compromised Pdx1 expression in beta-cells is tightly linked to hyperglycemia and loss of cellular identity [86]. Consequently, most studies have investigated the impact of either nutrient restriction or maternal high-fat feeding on Pdx1 expression in the offspring. In rodent studies, both lead to compromised expression in the pancreas/islets of offspring (Table 1).

Both uterine artery ligation and exposure to a maternal low-protein diet lead to a permanent suppression of Pdx1 in the offspring [26,33] irrespective of whether restricted nutrition leads to IUGR [20]. Interestingly, in those exposed to uterine artery ligation, beta-cell mass is unchanged at birth despite a 50% decrease in Pdx1 expression in fetal life [33]. Importantly, this permanent suppression of Pdx1 across the life course may offer an explanation for why offspring exposed to restricted nutrition are unable to expand their beta-cell mass in response to a gain in adiposity and insulin resistance in later life. It will be important to determine whether Pdx1 expression is different between male and female offspring exposed to restricted nutrition and whether this mediates sex-specific differences in T2D susceptibility.

In the case of exposure to maternal high-fat feeding throughout pregnancy and lactation, Pdx1 expression is reduced in islets of adult male offspring [66,74,82]. This effect also persists in the following (F2) generation [74]. Interestingly, in contrast to nutrient restricted offspring, beta-cell mass is increased in these offspring despite lower levels of Pdx1 [74,82]. Given that these studies investigated Pdx1 in offspring that were already metabolically compromised, it remains unclear whether compromised expression is present in offspring islets before the onset of metabolic dysfunction and is, therefore, programmed by maternal caloric excess or whether it is merely a consequence of declining islet function in these offspring. Whilst evidence for the former is present in the non-human primate model of maternal high-fat feeding; Pdx1 expression is not altered in fetal pancreas at gestational day 130 (early third trimester) [46], whether the same occurs in rodents is yet to be determined.

Thus far, only one study has compared Pdx1 expression in male versus female offspring. The authors found that in mice exposed to a maternal high-fat diet, Pdx1 expression was decreased in males but unchanged in females at four months of age [66]. The presence of this sex-specific difference in expression is likely because only male offspring displayed increased adiposity and were glucose intolerant at this age. In contrast, female offspring remained metabolically healthy [66].

### 4.2. The Impact of Restricted Nutrition versus Caloric Excess on Mitochondrial Metabolism and Oxidative Stress

Mitochondrial metabolism plays a central role in regulating insulin release from beta-cells. Mitochondria are the main source of ATP (energy) which together with other mitochondrial factors accomplish the coupling of glucose metabolism to insulin secretion [87]. Importantly, it has been shown that islet mitochondrial function in the offspring is sensitive to programming by the intrauterine and early life nutritional milieu (Table 1). Islets from offspring exposed to uterine artery ligation produce less ATP in response to 16.7mM glucose; an effect that is already present at one week of age and declines further with age [88]. A similar pattern is also observed for the activities of complex I and III of the mitochondrial electron transport chain and expression of mitochondrial-encoded genes resulting in decreased glucose- and leucine (mitochondrial fuel)-stimulated insulin secretion [88].

Mitochondrial dysfunction is frequently associated with oxidative stress, a phenomenon caused by an imbalance between the production and clearance of reactive oxygen species (ROS) in cells. In beta-cells, the production of ROS such as superoxide anions and hydrogen peroxide is coupled to glycolytic and respiratory metabolism [89]. These cells, however, are particularly vulnerable to oxidative stress due to their low levels of antioxidant enzymes, which is the first line of defense against excessive ROS levels [90]. Indeed, islets from offspring exposed to uterine artery ligation also show signs of oxidative stress (inferred from the presence of 4-Hydroxynonenal-protein adducts) from one week of age [88]. This is despite having higher levels of the mitochondrial antioxidant enzyme, manganese superoxide dismutase compared to controls [88], suggesting that whilst islets are able to mount an antioxidant defense, the system is likely overwhelmed by ROS.

T2D develops more rapidly in offspring growth restricted by uterine artery ligation compared to those exposed to maternal protein restriction; the former are diabetic at three months of age whereas at this same age, the latter have normal glucose tolerance. Despite this difference, protein-restricted offspring have compromised ATP production in response to glucose; an effect which is present in both males and females [21]. Surprisingly, glucose-stimulated insulin secretion was preserved in islets with female offspring secreting even more insulin than males in response to 16.7mM glucose. Expression of the mitochondrial transcription factor, Tfam, which is essential for mitochondrial DNA transcription and maintenance was decreased in female but not male islets. However, this did not have an overall effect of reducing the expression of mitochondrial-encoded genes [21]. In contrast, islets from male offspring exposed to a maternal low-protein diet produced more ROS in response to both low and high glucose levels [21], highlighting the complexities involved in interpreting the effects of compromised nutrition and growth on the mechanisms regulating islet function.

Exposure to a maternal obesogenic diet also leads to compromised mitochondrial function in offspring islets. However, timing of this nutritional insult and how severely the metabolic health of dams are compromised during pregnancy appears to determine whether or not there are sex-specific differences in susceptibility. We recently investigated mitochondrial function in islets from metabolically healthy adult mouse offspring (eight weeks of age) exposed to maternal obesity from before and throughout pregnancy and lactation. In this model, dams are heavier, fatter and glucose intolerant during pregnancy [91]. Islets from female offspring displayed increased mitochondrial respiration and density as well as increased expression of both mitochondrial- and nuclear-encoded components of the electron transport chain [65]. Not surprisingly, glucose- and mitochondrial fuel-stimulated insulin secretion is also increased in these offspring. Male offspring, however, have compromised mitochondrial respiration characterized by decreased ATP synthesis-driven respiration and increased “uncoupled” respiration but unlike in females, insulin secretion, mitochondrial density and the expression of genes encoding components of the electron transport chain are unaffected [65]. Upon stimulation with glucose, islets from both male and female offspring of obese dams produced more ROS compared to controls, however, only females had increased expression of antioxidant enzymes suggesting that islets from male offspring may be more vulnerable to oxidative stress [65]. A similar phenotype is also observed in the study by Yokomizo et al. where maternal high-fat feeding commences from conception. In this model, dams have increased body weight and higher fasting blood glucose levels during pregnancy. At four months of age, only islets from male offspring have signs of oxidative stress (measured by intensity of 8-hydroxy-2′-deoxyguanosine) and increased expression of Nox4 and gp91phox, which are involved in superoxide production [66]. It should be noted that at this age, male (and not female) offspring of high fat diet-fed dams have increased adiposity and impaired glucose tolerance compared to controls, however, the impact on glucose-stimulated insulin secretion in islets was not investigated.

In contrast, in the model used by Theys and colleagues, rats fed an obesogenic diet from conception have comparable body weight to controls throughout pregnancy except just before delivery when high fat diet-fed dams were lighter than controls [92]. In this study, dams were fed a diet with a much lower fat content compared to the study by Yokomizo et al. (23% versus 62% in [66]). Interestingly, at three months of age, islet function is already severely compromised in these offspring; both male and female offspring showed a loss of response to glucose, i.e., insulin secretion was not different between low and high glucose conditions [92]. In line this with finding, there was also no increase in glucose-stimulated ATP production. Basal ATP content was also reduced in offspring islets. Given that mitochondrial DNA content and the expression of Tfam and mitochondrial-encoded genes are unaffected in these islets, the mechanisms driving lowered ATP production remains to be determined.

### 4.3. Maternal Protein Restriction Leads to Reduced mTOR Signaling in Offspring Islets

Mammalian target of rapamycin (mTOR) is a nutrient-responsive kinase that exists as two functionally and structurally distinct complexes: mTOR complex 1 (mTORC1) and mTOR complex 2 (mTORC2). mTOR signaling plays a key role in integrating hormonal and nutritional stimuli to regulate cellular metabolism, survival and growth. mTOR is, therefore, integral for the maintenance of beta-cell development and function (reviewed in [93]). Given its critical role in nutrient-sensing and beta-cell homeostasis, studies have investigated whether the nutritional milieu experienced during fetal life leads to programming of altered mTOR signaling in the longer term and its effect on islet function. This has been investigated in the context of exposure to maternal protein restriction, which showed a persistent decrease in mTOR protein and/or mTOR activity, irrespective of whether offspring were growth restricted at birth or whether beta-cell mass is altered [18,20] (Table 1). Specifically, maternal protein restriction without IUGR leads to reduced mTORC1 activity (measured by phosphorylation of ribosomal protein S6 at Ser240) at birth when offspring also have a reduced beta-cell/pancreas ratio (beta-cell fraction). This effect persists in the offspring at three months of age despite a normalization of beta-cell mass. Importantly, transient activation of mTORC1 in beta-cells of protein-restricted offspring in the last week of pregnancy is able to rescue glucose intolerance in adult life and the defect in beta-cell fraction at birth by normalizing beta-cell proliferation in these mice [20]. In contrast, although IUGR offspring also have reduced beta-cells at birth, mTOR protein abundance is unchanged until after weaning with levels decreased at both 30 and 130 days of age (along with decreased beta-cell mass). However, whether mTOR activity is also altered in these offspring is unknown [18].

Islet mTOR protein and/or activity is also sensitive to the timing of protein restriction; when this is confined only to the last week of pregnancy, there is no impact on both mTOR protein abundance and phosphorylation of ribosomal protein S6 at Ser240 in the islets of three-month-old adult offspring. Whilst it is not known whether beta-cell function and mass are preserved at this age, glucose-stimulated insulin secretion is impaired and beta-cell mass increased in male offspring at 12 months of age [19].

### 4.4. Epigenetic Changes Underlying the Developmental Origins of Pancreatic Islet Dysfunction

Epigenetics involves a complex array of mechanisms including DNA methylation, histone modifications and microRNAs (miRNAs) that act to interpret the genome in a cell type-specific way. The rapid rise in T2D incidence over the past decades has highlighted the role of epigenetics (in addition to genetics) in contributing to disease risk. Thus far, only a small number of studies have focused on the role of epigenetics in mediating changes in gene expression and, therefore, function that is observed in islets of offspring exposed to compromised early life nutrition. However, we anticipate this to be an area of research with rapid growth in the coming years.

### 4.5. The Impact of Restricted Nutrition on DNA Methylation and Histone Modifications

DNA methylation is a major component of the mammalian epigenome and the only one for which inheritance through multiple cell divisions has been demonstrated. It, therefore, serves an essential function for stabilizing gene expression patterns throughout life [94]. DNA methylation can also be altered in response to environmental factors present in both pre- and postnatal life leading to cellular dysfunction and disease. For example, DNA methylation and chromatin accessibility of regulatory regions is altered in pancreatic islets from T2D donors with corresponding changes in gene expression and islet function [95,96,97,98]. Importantly, environmentally driven changes in DNA methylation can be maintained throughout life. Thus, aberrant epigenetic changes that result from in utero exposure to poor maternal nutritional states, e.g., undernutrition or obesity can have long-lasting effects on offspring health.

To date, our knowledge of such changes has been limited to the impact of IUGR on DNA methylation and histone modifications in islets. In particular, exposure to uterine artery ligation in fetal life leads to genome-wide changes in DNA methylation at intergenic (and not promoter) regions in islets from seven-week-old offspring [35]. At this age, fasting hyperglycemia has not yet emerged, however, islets already have compromised function [88]. Whole-genome histone modification maps of islets from two and 10-week-old offspring (using the same model as above) to define active promoters, transcriptionally silent chromatin and active enhancers also identified that the majority of differential histone marks were located in intergenic regions [32]. To better understand how DNA methylation contributes to the mechanisms underpinning T2D, it will be important to assign these sites of gene regulation, e.g., enhancers to their target gene(s). The latter is particularly challenging given that enhancers are often separated from the target genes they regulate by hundreds of thousands of base pairs.

In addition to a genome-wide approach, studies have also focused on identifying epigenetic dysregulation at specific candidate sites. Using the same model as above, Park and colleagues showed progressive changes in epigenetic regulation of Pdx1. This is observed through a gain in promoter methylation, which is present in offspring islets at six months of age but not at two and seven weeks. Additionally, there is also early and progressive changes in the acetylation and methylation of histones at the Pdx1 promoter. Interestingly, this is present in fetal life and with larger differences at two weeks and six months of age [34]. Consequently, there is a decline in Pdx1 expression in the islets of IUGR offspring with age, resulting in a complete absence of Pdx1 in three-month-old rats [33].

IUGR also leads to epigenetic changes that alter the expression of hepatocyte nuclear factor 4-α (HNF4α) in islets from three and 15-month-old rat offspring [99]. HNF4α is a transcription factor required for beta-cell differentiation and glucose homeostasis, and consequently has been linked to development of a number of forms of T2D. Specifically, maternal protein restriction during pregnancy and lactation leads to epigenetic silencing at the HNF4α enhancer region, which weakens the interaction with its promoter, resulting in a permanent reduction in gene expression. Furthermore, progressive epigenetic silencing of the entire HNF4α locus in islets in response to ageing is more pronounced in rats exposed to poor maternal diet. Importantly, these epigenetic changes precede T2D development in offspring, which occurs at 17 months of age and, therefore, represents a mechanistic basis for the cellular “memory” linking maternal diet to the development of T2D in the offspring [99]. It will be interesting to determine whether, like Pdx1, these changes are already present in fetal life.

### 4.6. The Impact of Restricted Nutrition on miRNA Expression

In addition to DNA methylation and histone modifications, miRNAs also have a central role to play in the development and function of beta-cells (reviewed in [100]). MiRNAs are small (~19–23 nucleotides) non-coding RNAs involved in post-transcriptional gene regulation and are, therefore, fundamental for cellular identity and function. So far, three studies have investigated the impact of maternal protein restriction on miRNA expression in offspring islets using an unbiased PCR-based array [20,26,60]. Alejandro et al. identified 14 miRNAs that were differentially expressed between offspring groups; two increased and 12 reduced. These measures were performed in three-month-old offspring who displayed glucose intolerance and impaired glucose-stimulated insulin secretion. Importantly, maternal protein restriction did not lead to IUGR in this model [20]. It is, therefore, not surprising that none of these same miRNAs were differentially expressed in islets from adult offspring who were growth restricted in utero; Su and colleagues showed that a different suite of 20 miRNAs were dysregulated in response to maternal protein restriction-induced IUGR [60]. Follow-up experiments focused on miR-15b, which is highly conserved amongst vertebrates and is up-regulated in IUGR islets, identified reduced expression of its target genes cyclin D1 and D2 at the protein level. Furthermore, inhibition of miR-15b in vitro was able to rescue the defective beta-cell proliferation and insulin secretion observed in offspring islets [60].

In contrast to these studies, which investigated miRNAs in the islets of adult offspring, Zhang et al. determined miRNA expression in whole pancreas from IUGR offspring in mid- and late-gestation and identified 31 miRNAs with increased and 54 miRNAs with decreased expression compared to controls [26]. Curiously, differences in miRNA expression are not maintained across gestation; most miRNAs were differentially expressed only at one fetal time point. Furthermore, given differences in the IUGR phenotype, tissue type and time points across the models, it is not surprising that there were no differentially expressed miRNAs in common between the studies. The expression of two miRNAs (miR-542-3p and miR-342-5p), however, are altered in response to IUGR. miR-542-3p is up-regulated in the fetal pancreas in mid- and late-gestation [26] but is decreased in the islets of adult offspring [60]. The expression of miR-342-5p is higher both in the pancreas of IUGR fetuses at embryonic day 19 [26] and in islets of three-month-old non-IUGR offspring [20]. It will be interesting to determine whether the presence of inflammatory markers is also increased in these offspring given that the expression of this miRNA is up-regulated in human islets in response to cytokine exposure [101]. It is important to note that whilst the studies by Alejandro et al. [20] and Zhang et al. [26] used comparable fold-change cut-offs for analysis (≥1.4 for up-regulated miRNAs and ≤0.55 for down-regulated miRNAs), the study by Su et al. showed only a modest change in up-regulated expression ranging from 1.1 to 1.9 fold [60].

Studies have also taken a candidate approach when screening for differentially expressed miRNAs. For example, Dumortier et al. measured the expression of miR-375, an islet-abundant miRNA with multiple roles in beta-cell function and found its expression increased in the pancreas of IUGR fetuses at 21 days gestation. This change persists in offspring islets at three months of age. Expression of pyruvate dehydrogenase kinase 1, a target of miR-375, was reduced at the mRNA and protein level [63]. Furthermore, in addition to the miRNAs identified by PCR-array, Alejandro and colleagues also measured the expression of miR-7 and miR-199a-3p, which regulate mTOR given that, as discussed above, exposure to maternal protein restriction leads to decreased insulin secretion that is due, in part, to reduced mTOR signaling. Expression of both miRNAs were increased in the islets of adult offspring [20]. The expression of miR-199a-3p has also been shown to be increased in islets from diabetic db/db mice but its expression is not yet altered in these mice in the pre-diabetic state [102].

Whilst some progress has been made to delineate epigenetic mechanisms driving islet dysfunction and T2D risk in offspring exposed to a nutrient-restricted intrauterine environment, there is still plenty of room for growth in this field especially in defining epigenetic networks that are driving sex-specific differences in islet function and which are also cell type-specific. Moreover, given that different environmental stimuli such as glucose, fatty acids and cytokines can regulate DNA methylation and miRNA expression [102,103,104,105,106,107], it is key that we understand which changes are programmed by the in utero environment and maintained throughout life, irrespective of the postnatal environment versus those that manifest as a result of declining metabolic health. This knowledge is equally important in the context of exposure to maternal caloric excess especially given that the impact of maternal obesity on the epigenetic landscape of offspring islets is still unknown.

## 5. Conclusions and Future Directions

It is clear from studies highlighted in this review that fetal malnutrition (encompassing exposure to either restricted nutrition or to an obesogenic diet) has long-lasting effects on islet-cell mass and function. Importantly, the decline in islet function and mass is not solely in response to pre-existing obesity and insulin resistance in adult offspring. Rather, there is inherent vulnerability in these islets resulting in their inability to successfully compensate when faced with increased functional demand.

Given this knowledge, it is key that we define the mechanisms mediating compromised islet function to better understand how T2D develops in those affected by malnutrition during fetal development. Whilst some progress has been made in this area, looking to the future, we believe that the field will greatly benefit from studies that move beyond a candidate approach to epigenetic and gene expression analyses toward genome-wide investigations. Importantly, the availability of next-generation sequencing technologies will enable the mapping of omics data to metabolic traits and will also allow for the characterization of novel mechanisms, for example, by combining data of transcription factor networks, chromatin state and gene transcription to generate an integrated map of gene regulation in islet cells. Furthermore, given that pancreatic islets consist of several endocrine cell types, the cell-specific pathways that are contributing to the functional phenotype should also be defined.

Finally, it is important to state that underpinning all of this research in animal models is the significant need to ensure that the mechanisms identified are also relevant to islet dysfunction and T2D pathogenesis in humans. This includes defining the outcomes relating to the interaction between an underlying genetic predisposition (to T2D) and exposure to in utero environmental drivers.

## Figures and Tables

**Table 1:** Similarities and differences in the mechanisms identified in offspring exposed to restricted nutrition versus caloric excess. *Red arrow: Decreased; Blue arrow: Increased; Black arrow: No change. Gray shading indicates data is not available*.

Environmental Exposure	Pdx1 Expression		Mitochondrial Metabolism +		Oxidative Stress 🡪		Functional Outcome		mTOR Abundance/Activity			
	*Fetal Life*	*Adult Life*	*Adult Life*						*Birth*		*Adult Life*	
Intrauterine growth restriction (due to maternal protein restriction or uterine artery ligation)	🡫 *(26, 33)*	🡫 *(26, 33)*	🡫 🡫	glucose-stimulated ATP *(87)* expression of mitochondrial-encoded genes *(87)*	🡩	oxidative stress *(87)*	🡫	glucose and mitochondrial-fuel stimulated insulin secretion *(87)*	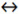	mTOR protein *(18)*	🡫	mTOR protein *(18)*
			Males and females:		Males only:		Males and females:					
			🡫	glucose-stimulated ATP *(21)*	🡩	reactive oxygen species *(21)*	🡩	glucose-stimulated insulin secretion *(21)*				
			Females only:									
			🡫 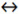	expression of Tfam *(21)* expression of mitochondrial-encoded genes *(21)*								
Maternal protein restriction without intrauterine growth restriction		🡫 *(20)*							🡫	mTOR activity *(20)*	🡫	mTOR activity *(20)*
Maternal caloric excess		Males: 🡫 *(65,73,81)* Females: 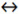 *(65)*	*Exposure to a high-fat, high-sugar diet from before and throughout pregnancy and lactation (64):*									
			Females:		Males and females:		Females:					
			🡩 🡩 🡩	mitochondrial respiration expression of mitochondrial-encoded genes mitochondrial density	🡩	reactive oxygen species	🡩	glucose and mitochondrial-fuel stimulated insulin secretion				
			Males:				Males:					
			🡫 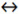 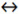	mitochondrial respiration expression of mitochondrial-encoded genes mitochondrial density			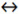	glucose and mitochondrial-fuel stimulated insulin				
			*Exposure to a high-fat diet from conception and throughout pregnancy and lactation (65):*									
					Males but not females:							
					🡩	oxidative stress						
					🡩	expression of genes involved in superoxide production						
			*Exposure to a high-fat diet from conception and throughout pregnancy and lactation (91):*									
			🡫 🡫	glucose-stimulated ATP basal ATP content			🡫	glucose stimulated insulin secretion				
			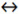 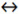 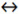	mitochondrial DNA content expression of Tfam expression of mitochondrial-encoded genes								
